# Erythromycin Suppresses the Cigarette Smoke Extract-Exposed Dendritic Cell-Mediated Polarization of CD4^+^ T Cells into Th17 Cells

**DOI:** 10.1155/2020/1387952

**Published:** 2020-01-21

**Authors:** Jifeng Liu, Xiaoning Zhong, Zhiyi He, Jianquan Zhang, Jing Bai, Guangnan Liu, Yi Liang, Leilei Ya, Xianglin Qin

**Affiliations:** ^1^Department of Respiratory Disease, First Affiliated Hospital of Guangxi Medical University, Nanning 530021, China; ^2^Department of Respiratory Disease, Second Affiliated Hospital of Guangxi Medical University, Nanning 530007, China

## Abstract

Cigarette smoke is a major effector of chronic obstructive pulmonary disease (COPD), and Th17 cells and dendritic cells (DCs) involve in the pathogenesis of COPD. Previous studies have demonstrated the anti-inflammatory effects of macrolides. However, the effects of macrolides on the cigarette smoke extract- (CSE-) induced immune response are unclear. Accordingly, in this study, we evaluated the effects of erythromycin (EM) on CSE-exposed DCs polarizing naïve CD4^+^ T cells into Th17 cells. DCs were generated from bone marrow-derived mononuclear cells isolated from male BALB/c mice and divided into five groups: control DC group, CSE-exposed DC group, CD40-antibody-blocked CSE-exposed DC group, and EM-treated CSE-exposed DC group. The function of polarizing CD4^+^ T cells into Th17 cells induced by all four groups of DCs was assayed based on the mixed lymphocyte reaction (MLR) of naïve CD4^+^ T cells. CD40 expression in DCs in the CSE-exposed group increased significantly compared with that in the control group (*P* < 0.05). The Th17 cells in the CSE-exposed DC/MLR group increased significantly compared with those in the control DC/MLR group (*P* < 0.05). Moreover, Th17 cells in the CD40-blocked CSE-exposed DC/MLR group and EM-treated CSE-exposed DC/MLR group were reduced compared with those in the CSE-exposed DC/MLR group (*P* < 0.05). Thus, these findings suggested that EM suppressed the CSE-exposed DC-mediated polarization of CD4^+^ T cells into Th17 cells and that this effect may be mediated through inhibition of the CD40/CD40L pathway.

## 1. Introduction

Smoking is a major cause of chronic nonspecific inflammation in chronic obstructive pulmonary disease (COPD) [1, 2]. Freeman et al. [3] showed that in patients with COPD, CD40 expression in dendritic cells (DCs) in the lung tissue was high, regardless of the Global Initiative for Chronic Obstructive Lung Disease stage. *In vitro* studies using bone marrow-derived and monocyte-derived immature DCs exposed to varying doses of nicotine and cigarette smoke extract (CSE) [4–6] have yielded contrasting results with respect to their effects on the function of DCs. Thus, DCs may play key roles in bridging innate and adaptive immunity via direct cell-cell interactions [7–9]. DCs induce CD4^+^ T cells to differentiate into Th1 cells via the CD40/CD40L pathway, and this process can be stimulated by interleukin- (IL-) 12 and interferon-*γ* [10–12]. DCs crosstalk with CD4^+^ T cells through the CD80/CD86 and CD28 pathways and secretion of IL-2, thereby promoting the differentiation of CD4^+^ T cells into regulatory T cells (Tregs) [13–16]. Several studies have suggested that DCs crosstalk with CD4^+^ T cells via the CD40/CD40L pathway and produce IL-6 and IL-23, which then mediate the development of Th17 cells by upregulating retinoic acid receptor-related orphan nuclear receptor *γ* (ROR*γ*t) mRNA in CD4^+^ T cells [17, 18]. At the same time, several studies have verified that DCs induced Th1, Th17, and Tc1 cell differentiation via the CD40/CD40L pathway in mice with emphysema exposed to cigarette smoke [19–21].

Recent studies have shown that macrolides suppress the expression of costimulatory molecules on DCs and exhibit anti-inflammatory effects by reducing the activity of phosphoinositide 3-kinase (PI3K), p38 mitogen-activated protein kinase (p38 MAPK), and nuclear factor-*κ*B (NF-*κ*B) [22, 23]. Moreover, macrolides have been shown to suppress the expression of costimulatory molecules on DCs [24]. Yasutomi et al. [25] showed that erythromycin could suppress the expression of costimulatory molecules on human monocyte-derived DCs which are induced by lipopolysaccharides, but erythromycin did not have the effect on DCs induced by peptidoglycan. This study suggested that the modulating-DC effects of EM depend on the nature of pathogens. Macrolides also suppress the Th17 response and inflammation in noncystic fibrosis bronchiectasis [26, 27]. Previous studies have shown that erythromycin (EM) reduces inflammation by suppressing the p38 MAPK and NF-*κ*B pathways, induced by CSE. Tan et al. [28] found that EM suppresses IL-17 and IL-23 associated with Th17 cells in the peripheral blood and induces sputum in patients with stable COPD. Interestingly, Bai et al. [29] showed that EM enhances CD4^+^Foxp3^+^ Treg numbers in rats exposed to cigarette smoke. Thus, EM may affect inflammation in Th17 cells induced by DCs in the context of COPD associated with cigarette smoke exposure. However, the mechanisms through which CSE promotes CD4^+^ T cell differentiation into Th17 cells following induction by DCs and whether EM has any effects on the cigarette smoke extract-exposed DC-mediated CD4^+^ T cell differentiation remain to be elucidated, which involve in the effect of nicotine and tar in CSE.

Accordingly, in this study, we evaluated the effects of CSE on the expression of the costimulatory molecules CD40 and CD86, expressed by immature bone marrow-derived dendritic cells (imDCs), following exposure to CSE. Additionally, we examined changes in the ability of DCs to polarize CD4^+^ T cells into Th17 cells in a mixed lymphocyte reaction (MLR) induced by CSE exposure and EM treatment [25, 30].

## 2. Materials and Methods

### 2.1. Reagents

Recombinant murine granulocyte-macrophage-colony-stimulating factor (GM-CSF) and recombinant murine IL-4 were obtained from PeproTech (London, UK). EM was obtained from Sigma-Aldrich (St. Louis, MO, USA). TRIzol and SuperScript II were obtained from Invitrogen (CA, USA). SYBR Green Universal PCR Master Mix was obtained from ABgene (Hamburg, Germany). Purified NA/LE hamster anti-mouse CD40 (antagonist) antibodies were obtained from BD Pharmingen (San Diego, CA, USA). Monensin, ionomycin, phorbol myristate acetate, and dimethyl sulfoxide were obtained from Sigma-Aldrich. FBS (Qualified Australia Origin), RPMI-1640, serum-free medium, immunomagnetic beads (Dynabeads (DB) Mouse DC Enrichment Kit and DB Untouched Mouse CD4^+^ T cell kit) were obtained from Invitrogen. Fluorescent-labelled monoclonal antibodies (fluorescein isothiocyanate (FITC) hamster anti-mouse CD11c, phycoerythrin (PE) rat anti-mouse CD40, allophycocyanin (APC) rat anti-mouse CD86, FITC rat anti-mouse CD4, and PE rat anti-mouse IL-17A) and the BD Cytofix/Cytoperm Fixation/Permeabilization kit were obtained from BD Pharmingen. A Quantibody array kit (cat. no. QAM-TH17-1) was obtained from RayBiotech (Norcross, GA, USA). *β*-Actin and *RORγt* primers, specific to BALB/c mice, were obtained from Takara (Tokyo, Japan).

### 2.2. Experimental Animals

BALB/c mice (4–6 weeks old) were obtained from the Laboratory Animal Center of Guangxi Medical University (Nanning, China). All experiments were approved by the Guangxi Medical University Committee on the Use and Care of Animals.

### 2.3. Preparation of CSE

CSE was produced according to the method described by Li et al. [22]. Briefly, CSE was generated by the burning of commercially available Marlboro cigarettes (made under authority of Philip Morris Brands Sarl Switzerland by China Tobacco Hunan Industrial Co. Ltd.; tar content: 12 mg, amount of nicotine: 0.9 mg, and carbon monoxide content:12 mg) without filter which were smoked to 0.5 cm above the filter in a fume hood. CSE (10%) was prepared by bubbling the smoke from two cigarettes in 20 mL serum-free RPMI at a rate of 1 cigarette/min. The pH of the RPMI was adjusted to 7.4, and the optical density was determined at 350 nm (0.81 ± 0.03). The medium was filter-sterilized with a 0.45 *μ*m filter cartridge (25 mm Acrodisc; Pall, Ann Arbor, MI). The CSE was always prepared fresh on the day of the experiment. The patterns of absorbance at 320 nm showed little difference between different preparations of CSE. The control medium was prepared by bubbling air through 20 mL serum-free RPMI (pH 7.4) that was filter-sterilized as described above.

### 2.4. Generation of Bone Marrow DC Cultures with GM-CSF and IL-4

The method for generating bone marrow-derived imDCs was described previously by Inaba et al. and Kim and Diamond [31, 32], Briefly, after removing all muscle tissues with gauze from the femurs and tibias, the bones were placed in a 60-ram dish with 70% alcohol for 1 rain, washed twice with PBS; bone marrow mononuclear cells were prepared from bone marrow suspensions by depletion of red cells, which were cultured at a density of 1 × 106 cells/mL in a RPMI-1640 medium in the presence of GM-CSF (40 ng/mL) and IL-4 (10 ng/mL) [31, 32]. The medium was replenished every 2 days, and the nonadherent clusters and loosely adherent DC were collected after day 6 of culture and used for further studies. The harvested cells were isolated using a DB Mouse DC Enrichment Kit (Invitrogen). DCs were purified by magnetic bead cell sorting. More than 90% of the cells had high expression of CD11c, but the cells had lower expression of CD80, CD86, CD40, and MHC class II (data not shown).

### 2.5. Cell Activation

imDCs were prepared as described previously [31, 32]. DCs were first starved in a serum-free medium with or without exposure to CSE (1.5%) for 24 h [4, 33]. After washing, the cells were used as CSE-exposed DCs treated with purified NA/LE hamster anti-mouse CD40 and EM [25, 30]. DCs, in the absence of CSE stimulation treated with purified NA/LE hamster anti-mouse CD40 and EM, were evaluated as controls. All DCs were harvested, and the expression of CD40 and CD86 was detected by flow cytometry.

### 2.6. Mixed Lymphocyte Reaction (MLR)

DCs (from BALB/c mice) at day 6 were pretreated with CSE (1.5%) [5, 33]. The CSE-exposed DCs were mixed with CD4^+^ T cells (from C57BL/6 mice) in the presence or absence of CD40 antagonist antibodies (purified NA/LE hamster anti-mouse CD40; 10 *μ*g/mL) [30]. The blocking was carried out for 24 h, and other CSE-exposed DCs were treated with or without EM (100 *μ*g/mL) for 24 h according to our preliminary experiment (data not shown) and the study of Yasutomi et al. [25], which showed that EM could suppress the expression of CD40 and CD86 or cytokines. The MLR was conducted in round-bottom 96-well microtest plates in 0.2 mL serum-free medium. To monitor the MLR, CD4^+^ T cells were isolated from spleens using a DB Untouched Mouse CD4^+^ T cell Kit (Invitrogen) according to the manufacturer's protocol. The DCs were precultured with or without CSE in the presence or absence of CD40 antagonistic antibody for blocking for 30 min, followed by coculture with allogeneic naïve T cells in round-bottom 96-well plates in 0.2 mL medium/well (2 × 10^4^ DCs/2 × 10^5^ CD4^+^ T cells). Simultaneously, CD4^+^ T cells were exposed to CSE, CD40 antagonistic antibody, and EM. Cells not exposed to CSE served as controls. Twenty-four hours after coculture, cell proliferation was assessed by analyzing *RORγt* mRNA expression using real-time quantitative polymerase chain reaction (PCR). Th17 cells (CD4^+^ IL-17A^+^) were then evaluated by flow cytometry. The cocultured supernatants were collected, and the cytokine contents were determined.

### 2.7. Flow Cytometry

Cells were incubated with the indicated monoclonal antibodies. DCs were stained with FITC-conjugated anti-CD11c, PE-conjugated anti-CD40, and APC-conjugated anti-CD86 antibodies. T cells were stained with APC-conjugated anti-CD4 and FITC-conjugated anti-T-cell receptor V*α*2 or PE-conjugated anti-CD40L antibodies. For intracellular cytokine detection, T cells were incubated with phorbol-12-myristate-13-acetate (10^−7^ M), ionomycin (1 *μ*g/mL), and monensin (3 *μ*M) for 4 h, fixed, permeabilized, and stained with FITC-labeled APC-conjugated anti-CD4 and PE-labeled APC-conjugated anti-IL-17A antibodies. The cells were pretreated with BD Cytofix/Cytoperm. Staining was performed on ice for 30 min, after which the cells were washed with ice-cold phosphate-buffered saline containing 0.1% NaN_3_ and 0.5% bovine serum albumin (Sigma-Aldrich). Subsequently, the cells were analyzed on a FACSCalibur (BD Pharmingen). The specific mean fluorescence intensities were calculated by subtracting the isotype-matched control antibody fluorescence, and data were analyzed using CellQuest software (BD Pharmingen).

### 2.8. Analysis of ROR*γ*t mRNA Expression

Total RNA for MLR was isolated using an RNeasy Kit (Qiagen, Dusseldorf, Germany). First-strand cDNA was synthesized from 1 *μ*g total RNA using SuperScript II Reverse Transcriptase (Invitrogen). PCR amplification of *RORγt* and *β*-actin was conducted using specific primer sequences as follows: *RORγt*, TCTGCAAGACTCATCGACAAGG (sense) and CACATGTTGGCTGCACAGG (antisense); *β*-actin, ATCCACGAAACTACCTTCAA (sense) and ATCCACACGGAGTACTTGC (antisense).

### 2.9. RNA Isolation and Real-Time PCR

Total RNA was extracted from T cells in MLR assays using TRIzol (Carlsbad, CA, USA) according to standard protocols. Reverse transcription (RT) was performed using SuperScript II. For real-time RT-PCR, cDNA was utilized for evaluating the expression of *RORγt* and *β*-actin genes using SYBR GREEN on an ABI Prism 7500 Sequence Detection System (Applied Biosystems, Foster City, CA, USA). The PCR conditions were as follows: 50°C for 2 min, 95°C for 10 min, and 40 cycles of 95°C for 15 s and 60°C for 1 min. The expression of *RORγt* mRNA (%) was defined as the cycle threshold (CT) value of *RORγt* mRNA divided by the CT of *β*-actin mRNA times 1000.

### 2.10. Quantification of IL-17A and IL-17F Levels in Culture Medium

The protein concentrations of IL-17A and IL-17F in cell culture supernatants were quantified using Quantibody array kits (RayBiotech Inc.) according to the manufacturer's instructions. The signal was scanned with a laser scanner by Axon GenePix using the green channel (excitation frequency = 532 nm). The data was analyzed by QAM-TH17-1 (analysis software).

### 2.11. Statistical Analysis

Experimental results are expressed as means ± standard errors of the means. The results were tested statistically by unpaired two-tailed Student's *t*-tests or one-way analysis of variance, followed by Newman-Keuls test for comparing all pairs of groups of data. Analyses were performed using the SPSS statistical software (version 16), and results were considered statistically significant when the *P* values were less than 0.05.

## 3. Results

### 3.1. Flow Cytometric Detection of CD40 and CD86 Expression in DCs

**Figure 1:**
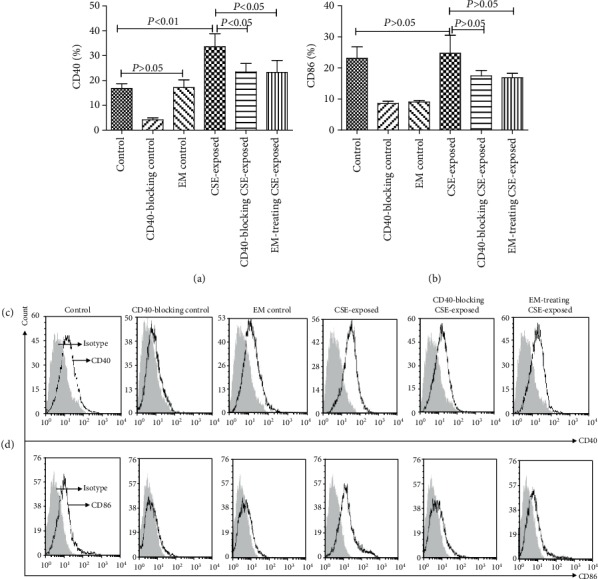
Effects of CSE and EM on the expression of costimulatory molecules (CD40 and CD86) in DCs. (a, c) Effects of CSE and EM on CD40 expression (*n* = 6). (b, d) Effects of CSE and EM on CD86 expression (*n* = 6). (The height of the bar chart represents the mean and the height of the error line indicates the standard errors of the means in the histograms.)

The expression of CD40 increased in the CSE-exposed group was compared with that in the control group (*P* < 0.05). No significant differences were observed in CD86 in the CSE-exposed group compared with the control group (*P* > 0.05). The expression of CD40 decreased in the CD40-blocked CSE-exposed group and the EM-treated CSE-exposed group compared with that in the CSE-exposed group (*P* < 0.05). However, no significant difference was noted with respect to the expression of CD86 between the CD40-blocked CSE-exposed, EM-treated CSE-exposed, and CSE-exposed groups (*P* > 0.05; Figure 1).

### 3.2. Th17 (CD4^+^IL-17A^+^) Detection by Flow Cytometry

**Figure 2:**
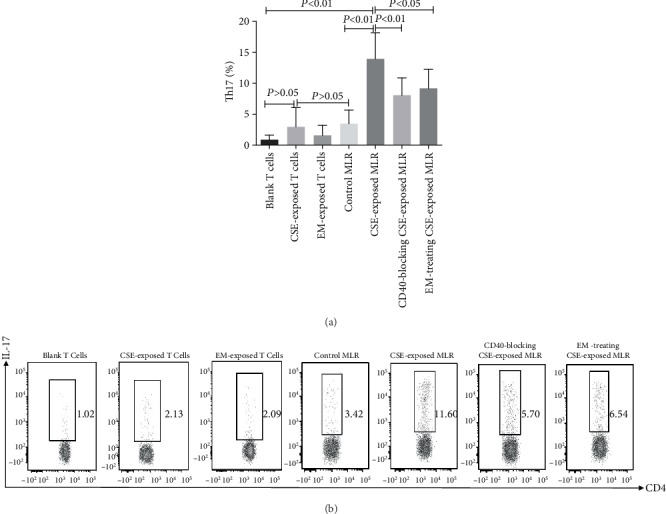
Effects of CSE and EM on DCs polarizing CD4^+^ T cells into Th17 cells. Numbers of Th17 cells in the various groups are shown (*n* = 6). (The height of the bar chart represents the mean and the height of the error line indicates the standard errors of the means in the histogram).

The number of Th17 (CD4^+^IL-17A^+^) cells was not increased in naïve CD4^+^ T cells after treatment with CSE, CD40 antagonist antibodies, or EM compared with the control group (*P* > 0.05). The number of Th17 (CD4^+^IL-17A^+^) cells in the CSE-exposed MLR group increased compared with that in the control MLR group (*P* < 0.05). The frequency of Th17 cells in the CD40-blocked CSE-exposed MLR group and EM-treated CSE-exposed MLR group was significantly reduced compared with that in the CSE-exposed MLR group (*P* < 0.05; Figure 2).

### 3.3. Expression of ROR*γ*t mRNA

**Figure 3:**
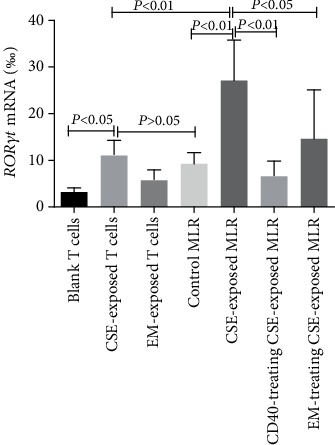
Effects of CSE and EM on the DC-induced expression of *RORγt* mRNA in the MLR. *RORγt* mRNA levels were evaluated by quantitative RT-PCR (*n* = 6). (The height of the bar chart represents the mean and the height of the error line indicates the standard errors of the means in the histogram.)

The mRNA level of *RORγt* increased in naïve CD4^+^ T cells after treatment with CSE compared with that in the control group (*P* < 0.05). However, treatment with CD40 antagonist antibodies and EM did not have similar effects (*P* > 0.05). The level of *RORγt* mRNA in the CSE-exposed MLR group was elevated compared with that in the control MLR group (*P* < 0.01), whereas those in the CD40-blocked CSE-exposed MLR group and EM-treated CSE-exposed MLR group were reduced significantly compared with that in the CSE-exposed MLR group (*P* < 0.05; Figure 3).

### 3.4. Determination of IL-17A and IL-17F Secretion by Antibody Microarray

**Figure 4:**
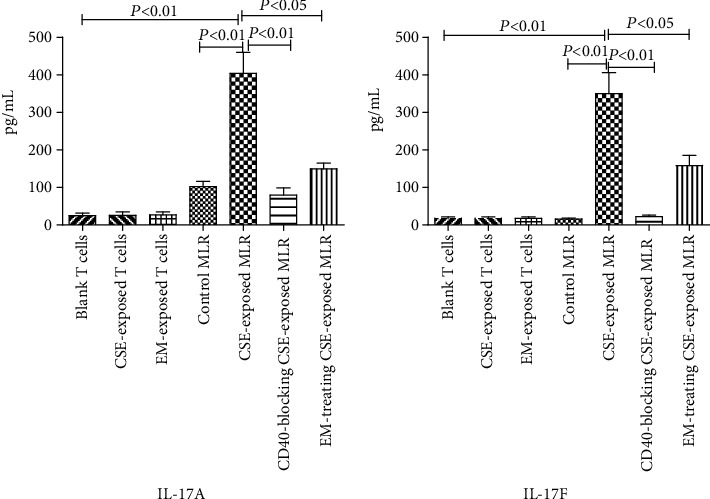
Effects of EM on the secretion of IL-17A and IL-17F in the CSE-exposed DC/MLR group. The levels of IL-17A and IL-17F were evaluated using Quantibody array kits (*n* = 6). (The height of the bar chart represents the mean and the height of the error line indicates the standard errors of the means in the histograms).

The levels of IL-17A and IL-17F were not significantly increased in the supernatants of the culture medium of naïve CD4^+^ T cells treated with CSE, CD40 antagonist antibodies, or EM when compared with that in the blank group (*P* > 0.05). Furthermore, the levels of cytokines in culture medium supernatants in the CSE-exposed MLR group were higher than those in the control MLR group (*P* < 0.01). Pretreatment with CD40 antagonist antibodies or EM decreased the secretion of IL-17A and IL-17F in the CSE-exposed DC/MLR group (*P* < 0.05; Figure 4).

## 4. Discussion

In this study, the expression of the costimulatory molecule CD40 increased on DCs following exposure to CSE. Hu et al. [5] showed that the costimulatory molecule CD40 was upregulated in imDCs when the cells were stimulated with an appropriate concentration of nicotine *in vitro*. CD4^+^ T cells differentiated into Th17 cells by directed transcription of the orphan nuclear receptor ROR*γ*t in CD4^+^ T cells. Th17 cells secrete IL-17A and IL-17F, which recruit neutrophils as effector cells [34]. Our current results showed that CSE-exposed DCs significantly induced CD4^+^ T cells to polarize into Th17 cells compared with unexposed DCs. Moreover, the levels of *RORγt* mRNA, IL-17A, and IL-17F increased in the MLR for CSE-exposed DCs. Additionally, pretreatment with antagonistic anti-CD40 antibodies decreased the ability of DCs to polarize CD4^+^ T cells into Th17 cells. *RORγt* expression and IL-17A and IL-17F levels increased following antagonistic anti-CD40 pretreatment. These results implied that DCs induced CD4^+^ T cell differentiation into Th17 cells via the crosstalk of the costimulatory molecule CD40 with CD40L on CD4^+^ T cells. Earlier studies have reported the involvement of IL-6 in CD4^+^ T cell polarization through a mechanism dependent on upregulation of *RORγt* transcription [4–6]. In the current study, we showed that the CSE could not induce the differentiation of CD4^+^ T cells into Th17 cells, although CSE stimulated an increase in *RORγt* mRNA levels in CD4^+^ T cells. Furthermore, the differentiation of Th17 cells has been shown to require robust antigenic stimulation provided by the augmented CD40L expression on T cells, which results in increased activation of DCs and production of IL-6 [3–6, 35]. Moreover, several studies have demonstrated that CD40-deficient DCs showed impaired development of Th17 cells in response to immunization with high-dose antigens [4–6, 33]. Taken together, these results suggest that CSE-exposed DCs may drive the differentiation of IL-17-producing CD4^+^ T cells by fostering CD40/CD40L crosstalk.

In this study, we showed that EM suppressed the expression of CD40 and reduced the frequency of Th17 cells polarized from CD4^+^ T cells by CSE-treated DCs. In a previous study, we found that EM inhibited the activation of p38 MAPK and NF-*κ*B in macrophages exposed to CSE [22]. Additionally, the costimulatory molecule CD40 is upregulated by activation of PI3K, p38 MAPK, and NF-*κ*B in DCs [36–39]. Recent studies have shown that macrolides suppress the expression of costimulatory molecules on DCs [24] and exhibit anti-inflammatory effects by reducing the activities of PI3K, p38 MAPK, and NF-*κ*B [23]. Furthermore, macrolides have been shown to suppress the Th17 response and inflammation in noncystic fibrosis bronchiectasis [26, 27]. Thus, EM may reduce DC-mediated polarization of CD4^+^ T cells to Th17 cells through suppression of the CD40/CD40L pathway. Smoking is the primary cause of COPD, and our studies showed that CSE-exposed DCs polarized CD4^+^ T cells to Th17 cells via the CD40/CD40L pathway. This phenomenon suggested the occurrence of crosstalk of DCs with CD4^+^ T cells via CD40/CD40L, thereby playing a crucial role in Th17 cell differentiation in COPD [2, 20]. Therefore, smoking and inflammation are closely linked in the COPD environment, which initiates the expression of CD40 on DCs to induce CD4^+^ T cell differentiation into Th17 cells. These Th17 cells are involved in the immune and inflammatory damage observed in COPD. Lee et al. [40] showed that nicotine of CSE could induce reactive oxygen species (ROS) cytotoxicity and heme oxygenase-1 (HO-1) could block the cytotoxic effects via the p38 MAP kinase, PI3 K, and NF-*κ*B signaling pathways. On the other side, HO-1 can influence DC function through effects on the p38 MAPK-CREB/ATF1 signaling pathway [41]. Whether the nicotine of CSE induces ROS and HO-1 involves in regulation of DCs exposed to CSE and COPD need to be studied. At the same time, erythromycin can reduce ROS of CSE-exposed macrophages [42]. Whether the erythromycin has an effect on HO-1 of CSE-exposed DCs, COPD may need to be further studied. Our current findings suggested that the EM suppresses the CD40/CD40L pathway and reduces the ability of the CSE-exposed DCs to polarize the CD4+ T cells to Th17 cells. Notably, EM suppresses inflammation of COPD by downregulating the p38 MAPK and NF-*κ*B signaling pathways stimulated by CSE [22, 43, 44]. Two other studies have shown that EM enhances CD4^+^Foxp3^+^ regulatory T cells in rats exposed to smoke, whereas the levels of IL-17 and IL-23 associated with Th17 were suppressed in the peripheral blood. This phenomenon induces sputum in patients with stable COPD [28]. Thus, macrolides may suppress Th-17-associated inflammation. However, the roles of CD40/CD40L in DC-mediated crosstalk and polarization of CD4^+^ T cells to Th17 cells involved in inflammatory damage in COPD in vivo have not yet been elucidated. Furthermore, the anti-inflammatory effects of macrolides induced by suppressing the CD40/CD40L pathway in DC-mediated crosstalk in COPD need to be studied in vivo.

## 5. Conclusions

In summary, our data demonstrated that CSE augmented CD40 expression by stimulating DCs. CSE could promote DCs to induce naïve CD4^+^ T cells to differentiate into Th17 cells. Blocking the CD40/CD40L pathway could reduce the number of naïve CD4^+^ T cells polarizing to Th17 cells as a result of exposure of DCs to CSE. In addition, our observations implied that EM may reduce the polarization of CD4^+^ T cells to Th17 cells by DCs, resulting in suppression of the CD40/CD40L pathway after exposure to CSE.

## Data Availability

We all considered the original data used to support the findings of this study might be currently under embargo. Requests for data, 6 months after publication of this article, will be considered by the corresponding author.

## References

[1] Vestbo J., Hurd S. S., Agustí A. G. (2013). Global strategy for the diagnosis, management, and prevention of chronic obstructive pulmonary disease: GOLD executive summary. *American Journal of Respiratory and Critical Care Medicine*.

[2] Brusselle G. G., Joos G. F., Bracke K. R. (2011). New insights into the immunology of chronic obstructive pulmonary disease. *The Lancet*.

[3] Freeman C. M., Martinez F. J., Han M. L. K. (2009). Lung dendritic cell expression of maturation molecules increases with worsening chronic obstructive pulmonary disease. *American Journal of Respiratory and Critical Care Medicine*.

[4] Aicher A., Heeschen C., Mohaupt M., Cooke J. P., Zeiher A. M., Dimmeler S. (2003). Nicotine strongly activates dendritic cell-mediated adaptive immunity: potential role for progression of atherosclerotic lesions. *Circulation*.

[5] Hu S. X., Sui H. X., Jin H. J. (2012). Lipopolysaccharide and dose of nicotine determine the effects of nicotine on murine bone marrow-derived dendritic cells. *Molecular Medicine Reports*.

[6] Vassallo R., Kroening P. R., Parambil J., Kita H. (2008). Nicotine and oxidative cigarette smoke constituents induce immune-modulatory and pro-inflammatory dendritic cell responses. *Molecular Immunology*.

[7] Banchereau J., Steinman R. M. (1998). Dendritic cells and the control of immunity. *Nature*.

[8] Liu Y. J. (2001). Dendritic cell subsets and lineages, and their functions in innate and adaptive immunity. *Cell*.

[9] Satpathy A. T., Wu X., Albring J. C., Murphy K. M. (2012). Re(de)fining the dendritic cell lineage. *Nature Immunology*.

[10] Heufler C., Koch F., Stanzl U. (1996). Interleukin-12 is produced by dendritic cells and mediates T helper 1 development as well as interferon-*γ* production by T helper 1 cells. *European Journal of Immunology*.

[11] Macatonia S. E., Hosken N. A., Litton M. (1995). Dendritic cells produce IL-12 and direct the development of Th1 cells from naive CD4^+^ T cells. *Journal of Immunology*.

[12] Stuber E., Strober W., Neurath M. (1996). Blocking the CD40L-CD40 interaction in vivo specifically prevents the priming of T helper 1 cells through the inhibition of interleukin 12 secretion. *Journal of Experimental Medicine*.

[13] Yamazaki S., Steinman R. M. (2009). Dendritic cells as controllers of antigen-specific Foxp3+ regulatory T cells. *Journal of Dermatological Science*.

[14] Tai X., Cowan M., Feigenbaum L., Singer A. (2005). CD28 costimulation of developing thymocytes induces *Foxp3* expression and regulatory T cell differentiation independently of interleukin 2. *Nature Immunology*.

[15] Pletinckx K., Döhler A., Pavlovic V., Lutz M. B. (2011). Role of dendritic cell maturity/costimulation for generation, homeostasis, and suppressive activity of regulatory T cells. *Frontiers in Immunology*.

[16] O’Sullivan B. J., Pai S., Street S. (2011). Immunotherapy with costimulatory dendritic cells to control autoimmune inflammation. *Journal of Immunology*.

[17] Iezzi G., Sonderegger I., Ampenberger F., Schmitz N., Marsland B. J., Kopf M. (2009). CD40-CD40L cross-talk integrates strong antigenic signals and microbial stimuli to induce development of IL-17-producing CD4+ T cells. *Proceedings of the National Academy of Sciences of the United States of America*.

[18] Perona-Wright G., Jenkins S. J., O'Connor R. A. (2009). A pivotal role for CD40-mediated IL-6 production by dendritic cells during IL-17 induction in vivo. *Journal of Immunology*.

[19] Kuang L. J., Deng T. T., Wang Q. (2016). Dendritic cells induce Tc1 cell differentiation via the CD40/CD40L pathway in mice after exposure to cigarette smoke. *American Journal of Physiology-Lung Cellular and Molecular Physiology*.

[20] Shan M., Cheng H.-F., Song L.-z. (2009). Lung myeloid dendritic cells coordinately induce T_H_1 and T_H_17 responses in human emphysema. *Science Translational Medicine*.

[21] Liang Y., Shen Y., Kuang L. (2018). Cigarette smoke exposure promotes differentiation of CD4^+^ T cells toward Th17 cells by CD40-CD40L costimulatory pathway in mice. *International Journal of Chronic Obstructive Pulmonary Disease*.

[22] Li M., Zhong X., He Z. (2012). Effect of erythromycin on cigarette-induced histone deacetylase protein expression and nuclear factor-*κ*B activity in human macrophages in vitro. *International Immunopharmacology*.

[23] Kobayashi Y., Wada H., Rossios C. (2013). A novel macrolide/fluoroketolide, solithromycin (CEM-101), reverses corticosteroid insensitivity via phosphoinositide 3-kinase pathway inhibition. *British Journal of Pharmacology*.

[24] Iwamoto S., Kumamoto T., Azuma E. (2011). The effect of azithromycin on the maturation and function of murine bone marrow-derived dendritic cells. *Clinical and Experimental Immunology*.

[25] Yasutomi M., Ohshima Y., Omata N. (2005). Erythromycin differentially inhibits lipopolysaccharide- or poly(I:C)-induced but not peptidoglycan-induced activation of human monocyte-derived dendritic cells. *The Journal of Immunology*.

[26] Fouka E., Lamprianidou E., Arvanitidis K. (2014). Low-dose clarithromycin therapy modulates Th17 response in non-cystic fibrosis bronchiectasis patients. *Lung*.

[27] Liu J., Zhong X., He Z. (2014). Effect of low-dose, long-term roxithromycin on airway inflammation and remodeling of stable noncystic fibrosis bronchiectasis. *Mediators of Inflammation*.

[28] Tan C., Huang H., Zhang J., He Z., Zhong X., Bai J. (2016). Effects of low-dose and long-term treatment with erythromycin on interleukin-17 and interleukin-23 in peripheral blood and induced sputum in patients with stable chronic obstructive pulmonary disease. *Mediators of Inflammation*.

[29] Bai J., Qiu S. L., Zhong X. N. (2012). Erythromycin enhances CD4^+^ Foxp3^+^ regulatory T-cell responses in a rat model of smoke-induced lung inflammation. *Mediators of Inflammation*.

[30] Karimi M. H., Ebadi P., Pourfathollah A. A., Moazzeni S. M. (2010). Tolerance induction by CD40 blocking through specific antibody in dendritic cells. *Iranian Journal of Allergy, Asthma, and Immunology*.

[31] Inaba K., Inaba M., Romani N. (1992). Generation of large numbers of dendritic cells from mouse bone marrow cultures supplemented with granulocyte/macrophage colony-stimulating factor. *Journal of Experimental Medicine*.

[32] Kim S. J., Diamond B. (2007). Generation and maturation of bone marrow-derived DCs under serum-free conditions. *Journal of Immunological Methods*.

[33] Mortaz E., Kraneveld A. D., Smit J. J. (2009). Effect of cigarette smoke extract on dendritic cells and their impact on T-cell proliferation. *PLoS One*.

[34] Ivanov I. I., McKenzie B. S., Zhou L. (2006). The orphan nuclear receptor ROR*γ*t directs the differentiation program of proinflammatory IL-17^+^ T helper cells. *Cell*.

[35] Katzman S. D., Gallo E., Hoyer K. K., Abbas A. K. (2011). Differential requirements for Th1 and Th17 responses to a systemic self-antigen. *Journal of Immunology*.

[36] Ardeshna K. M., Pizzey A. R., Devereux S., Khwaja A. (2000). The PI3 kinase, p38 SAP kinase, and NF-*κ*B signal transduction pathways are involved in the survival and maturation of lipopolysaccharide-stimulated human monocyte-derived dendritic cells. *Blood*.

[37] Makela S. M., Strengell M., Pietila T. E., Osterlund P., Julkunen I. (2009). Multiple signaling pathways contribute to synergistic TLR ligand-dependent cytokine gene expression in human monocyte-derived macrophages and dendritic cells. *Journal of Leukocyte Biology*.

[38] Doffek K., Chen X., Sugg S. L., Shilyansky J. (2011). Phosphatidylserine inhibits NF*κ*B and p38 MAPK activation in human monocyte derived dendritic cells. *Molecular Immunology*.

[39] Neves B. M., Cruz M. T., Francisco V. (2009). Differential roles of PI3-kinase, MAPKs and NF-*κ*B on the manipulation of dendritic cell T_h_1/T_h_2 cytokine/chemokine polarizing profile. *Molecular Immunology*.

[40] Lee H. J., Lee J., Min S. K. (2008). Differential induction of heme oxygenase-1 against nicotine-induced cytotoxicity via the PI3K, MAPK, and NF-kappa B pathways in immortalized and malignant human oral keratinocytes. *Journal of Oral Pathology & Medicine*.

[41] Al-Huseini L. M. A., Yeang H. X. A., Hamdam J. M. (2014). Heme oxygenase-1 regulates dendritic cell function through modulation of p 38 MAPK-CREB/ATF1 signaling. *Journal of Biological Chemistry*.

[42] Ma N., Deng T. T., Wang Q. (2019). Erythromycin regulates cigarette smoke-induced proinflammatory mediator release through Sirtuin 1-nuclear factor *κ*B axis in macrophages and mice lungs. *Pathobiology*.

[43] Duan M. C., Zhong X. N., Huang Y., He Z. Y., Tang H. J. (2011). Mechanisms and dynamics of Th17 cells in mice with cigarette smoke-induced emphysema. *Zhonghua Yi Xue Za Zhi*.

[44] He Z. Y., Ou L. M., Zhang J. Q. (2010). Effect of 6 months of erythromycin treatment on inflammatory cells in induced sputum and exacerbations in chronic obstructive pulmonary disease. *Respiration*.

